# Cyberchondria, but not preventive behavior, mediates the relationship between fear of COVID-19 and somatic burden: Evidence from Russia

**DOI:** 10.3389/fpsyt.2022.1018659

**Published:** 2022-09-26

**Authors:** Alena Zolotareva

**Affiliations:** School of Psychology, HSE University, Moscow, Russia

**Keywords:** somatic burden, fear of COVID-19, cyberchondria, preventive behavior, COVID-19 pandemic

## Abstract

**Background:**

There is extensive available research on the relationship between fear of COVID-19 pandemic and physical symptoms. This study was the first to examine the cyberchondria and COVID-19 preventive behavior as mediators of this relationship.

**Methods:**

A cross-sectional study was conducted from October to December 2021, during the fourth wave of the COVID-19 pandemic in Russia. The participants were 2,011 Russian-speaking volunteers aged 18 years and older. They completed questionnaires on somatic burden, cyberchondria, COVID-19 preventive behavior, and fear of COVID-19 pandemic. Mediation analysis was used to explore the mediating roles of cyberchondria and preventive behavior in the relationship between fear of COVID-19 and somatic burden.

**Results:**

Fear of COVID-19 positively predicted somatic burden, cyberchondria, and COVID-19 preventive behavior. Mediation analysis showed that the relationship between fear of COVID-19 pandemic and somatic burden was mediated by cyberchondria (effect = 0.08, bootstrapping SE = 0.01, bootstrapping 95% CI [0.08, 0.12]), but not COVID-19 preventive behavior (effect = 0.02, bootstrapping SE = 0.01, bootstrapping 95% CI [0.00, 0.05]).

**Conclusion:**

The findings suggest that cyberchondria had negative effects on somatic burden during the COVID-19 pandemic. The knowledge of the mediating role of cyberchondria may be used by health care workers when consulting persons with physical health complaints and psychosomatic disorders.

## Introduction

The COVID-19 pandemic has challenged the mental and physical health of the population in all countries. Many people have experienced a relapse of chronic and psychiatric diseases (1), residual physical and cognitive effects (2), emotional exhaustion (3), psychological distress (4), posttraumatic stress disorder (5), psychosomatic burden (6), stigmatization, discrimination (7), isolation, loneliness, and concerns about their health and future (8).

One of the most common effects of pandemic is fear of COVID-19, found in 18–45% of persons in the general population (9, 10). Numerous studies showed that fear of COVID-19 is triggered by feeling of isolation from others and those close friends and family members who are infected with the SARS-CoV-2 (11), weak basic beliefs in goodness and justice of the world around (12), chronic illnesses, perception of bad government response to a pandemic (13), media exposure, intolerance of uncertainty, health anxiety, perceived risk for loved ones, economic consequences, and health care systems overload (14). Females, younger adults, urban residents, divorcees, healthcare workers, quarantined persons, persons suspected of being infected, and persons with mental disorders were at increased risk for fear of COVID-19 (15).

### Fear of COVID-19, cyberchondria, and preventive behavior

Not surprisingly, fear of COVID-19 can greatly alter human behavior, especially as it relates to health and safety. Pre-pandemic research showed that more than 50% of Internet users receive medical information through online news, newspapers, and magazines (16). These trends worsened during the COVID-19 pandemic, triggering an increase in health anxiety and a search for information about a mysterious and frightening infection (17). Persons with a high fear of pandemic often search the Internet for information about COVID-19 disease, so fear of COVID-19 was closely associated with cyberchondria (18–20).

Because fear of COVID-19 can change health-related behavior, it can also influence adherence to preventive measures. Since the World Health Organization declared the COVID-19 pandemic, governments of various countries have developed preventive behaviors including hand hygiene, wearing facemasks, social distancing, working from home, and avoiding any non-essential local and international travel (21). Persons with chronic diseases (22), greater COVID-19 threat appraisal (23), sufficient knowledge about the pandemic (24), greater government trust (25), greater health literacy and access to COVID-19 information sources (26), greater positive perception of social media, e-government services and information (27) observed preventive behavior more frequently and intensively.

Fear of COVID-19 has activated preventive behavior (28–30). This trend is highlighted among vulnerable persons, such as pregnant females (31), older persons (32), and persons with mental disorders (33). With long-term observation of the relationship between fear of COVID-19 and preventive behavior, it is clear that this tendency may be significant during a severe, but not mild COVID-19 outbreak period (34).

### Effects of COVID-19 fear on somatic burden

In a broad sense, somatic burden occurs as a physiological reaction to strong emotional impressions. A person in a state of fear may have elevated blood pressure, pulse, and respiration, which decrease when the fear passes, but remain when a person experiences permanent or persistent fear and can not express it in emotion or behavior (35). Fear of COVID-19 refers to these permanent or persistent emotional disturbances due to the duration of dangerous and life-threatening circumstances, Therefore, persons who expressed greater fear of COVID-19 had more severe and frequent somatic symptoms (36–38).

### The present study

Obviously, the relationship between fear of COVID-19 and somatic burden can be mediated by various phenomena, but so far only the buffering effect of perceived social support has been studied (38). Considering the relationship between fear of COVID-19 and somatic burden, as well as the relationship between fear of COVID-19, cyberchondria, and preventive behavior, it has been suggested that cyberchondria and preventive behavior may mediate the relationship between fear of COVID-19 and somatic burden.

**Figure 1:**
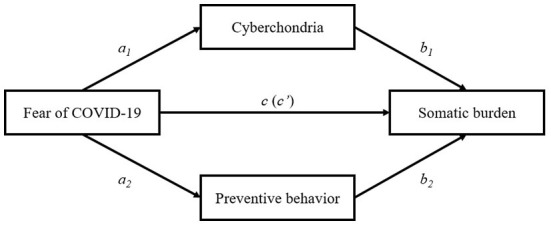
Proposed multiple mediation model; Direct and indirect effects of COVID-19 fear on somatic burden. c – total effect; c' – direct effect.

Based on previous empirical highlights (22, 37–39), it has been hypothesized that fear of COVID-19, cyberchondria, and preventive behavior would be positively associated with somatic burden (H1). Also, it seemed obvious to suggest that both cyberchondria and preventive behavior positively mediate the relationship between fear of COVID-19 and somatic burden (H2). This hypothesis was based on the relationship between cyberchondria and somatic burden found before the pandemic (40), and the fact that frequent primary health care attendance is typical for persons with somatic symptom disorder (41). The proposed multiple mediation model is presented in Figure 1.

## Materials and methods

### Sample and procedure

The data were collected in December 2021. The participants were recruited from a community sample with the support of the Russian survey company Anketolog. This company sent out letters to its subscribers inviting them to take part in the study. The only criterion was 18 years of age or older. Volunteers who responded to the invitation gave written informed consent, filled out a questionnaire, and received a financial reward for participating in the study. A sample consisted of 2,011 volunteers (1,335 females and 676 males) aged 18–80 years old (*M* = 40.91, *Mdn* = 40, *SD* = 10.57).

### Evaluation instruments

Somatic burden was evaluated with the Somatic Symptoms Scale-8 (SSS-8). The SSS-8 is an 8-item scale developed (42) and adapted into Russian (43) to measure somatic burden through eight somatic complaints. Symptoms are scored on a five-point response option from 0 (“not bothered at all”) to 4 (“bothered very much”). Cronbach's alpha was 0.83 in the current study.

Fear of COVID-19 was evaluated with the Fear of COVID-19 Scale (FCV-19S). The FCV-19S is a 7-item scale developed (44) and adapted into Russian (45) to measure psychological and physiological responses to fear associated with SARS-CoV-2 and COVID-19 pandemic. Responses range from 1 (“strongly disagree”) to 5 (“strongly agree”). Cronbach's alpha was 0.83 in the current study.

Cyberchondria was evaluated with the Cyberchondria Severity Scale-12 (CSS-12). The CSS-12 is a 12-item scale developed (46) and adapted (47) to measure excessive, compulsive, and distressing searches for health information on the Internet. Each statement is rated from 1 (“never”) to 5 (“always”). Cronbach's alpha was 0.95 in the current study.

Preventive behavior was evaluated with the COVID-19 Preventive Behavior Index (CPBI). The CPBI is a 10-item scale developed (21) to measure preventive behaviors aimed at reducing exposure to SARS-CoV-2 and COVID-19 pandemic. Responses range from 1 (“strongly disagree”) to 5 (“strongly agree”). In the current study, the CPBI was translated into Russian with a standardized procedure (48). The translated Russian version of the CPBI showed acceptable reliability (Cronbach's alpha was 0.86) and factor validity (χ^2^ = 492.655, df = 33, *p* < 0.001, TLI = 0.918, CFI = 0.940, RMSEA = 0.083 [0.077–0.090]).

### Analytic strategy

All data analyses were performed using jamovi 1.6.15 and IBM SPSS Statistics 27.0. Descriptive statistics were tested using means and standard deviations. Bivariate relations between measured variables were assessed with Pearson correlations. A *p*-value of 0.05 or lower was considered statistically significant. Mediation model was conducted using Hayes' Process with Model 4. It examined cyberchondria and preventive behavior as mediators in the relationship between fear of COVID-19 and somatic burden. Statistical significance of the indirect effects were established using the bootstrapping method based on 10,000 resamples. The indirect effect was considered significant if the confidence intervals were entirely above or below zero (49).

## Results

### Preliminary analysis

Table 1 presents descriptive statistics and Pearson's correlation coefficients for all measured variables. Somatic burden was positively related to fear of COVID-19, cyberchondria, and preventive behavior. Fear of COVID-19 was positively correlated with cyberchondria and preventive behavior. Finally, cyberchondria was positively associated with preventive behavior during the COVID-19 pandemic.

**Table 1:** Descriptive statistics and correlation matrix.

**Variable**	**M**	**SD**	**1**	**2**	**3**
1. Somatic burden	10.32	6.24			
2. Fear of COVID-19	16.43	5.25	0.27^***^		
3. Cyberchondria	21.53	9.26	0.31^***^	0.33^***^	
4. Preventive behavior	33.42	8.07	0.15^***^	0.46^***^	0.13^***^

*** p < 0.001.

### Mediation analysis

**Figure 2:**
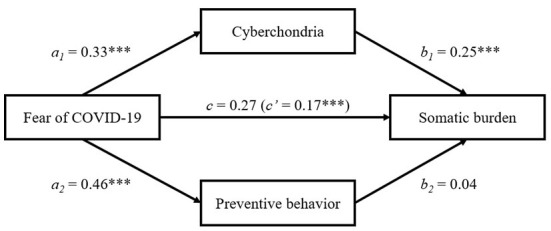
Mediation effects of cyberchondria and preventive behavior in the relationship between COVID-19 fear and somatic burden. All coefficients are standardized. c – total effect; c' – direct effect; ***p < 0.001.

A multiple mediation analysis was used to examine whether cyberchondria and preventive behavior mediate the relationship between fear of COVID-19 and somatic burden (Figure 2). There was a significant positive effect of COVID-19 fear on cyberchondria (*a*_1_: β = 0.33, *SE* = 0.04, *p* < 0.001), and on preventive behavior (*a*_2_: β = 0.46, *SE* = 0.03, *p* < 0.001). Cyberchondria (*b*_1_: β = 0.25, *SE* = 0.01, *p* < 0.001) was positively related to somatic burden, whereas preventive behavior (*b*_2_: β = 0.04, *SE* = 0.01, *p* = 0.115) had non-significant effect on somatic burden. The total effect of COVID-19 fear on somatic burden was significant (*c*: β = 0.27, *SE* = 0.03, *p* < 0.001), and after entering cyberchondria and preventive behavior into the model, the direct effect of COVID-19 fear on somatic burden remained significant (*c'*: β = 0.17, *SE* = 0.03, *p* < 0.001). Using the bootstrapping procedure (10,000 resamples), the analysis showed that indirect effect of COVID-19 fear on somatic burden through cyberchondria was significant (effect = 0.08, bootstrapping SE = 0.01, bootstrapping 95% CI [0.08, 0.12]), whereas indirect effect of COVID-19 fear on somatic burden through preventive behavior was non-significant (effect = 0.02, bootstrapping SE = 0.01, bootstrapping 95% CI [0.00, 0.05]).

## Discussion

This study was aimed to examine two research hypotheses: (H1) the relationship between fear of COVID-19, cyberchondria, and preventive behavior would be positively associated with somatic burden; (H2) cyberchondria and preventive behavior positively mediate the relationship between fear of COVID-19 and somatic burden. Firstly, the results of correlation analysis showed that somatic burden was positively correlated with fear of COVID-19, cyberchondria, and preventive behavior. This fully confirmed H1. Secondly, the results of mediation analysis indicated that the relationship between fear of COVID-19 and somatic burden was mediated by cyberchondria, but not preventive behavior. This partially supported H2.

These findings are consistent with previous highlights that somatic burden is associated with cyberchondria (40) and fear of COVID-19 (38). Cyberchondria was a positive mediator in the relationship between fear of COVID-19 and somatic burden, that is, when it grows, fear of COVID-19 leads to increased somatization. During the COVID-19 pandemic, cyberchondria was linked to many negative conditions including health anxiety, anxiety about a pandemic, depressive symptoms, obsessive-compulsive symptoms, and problematic usage of the Internet (50). Since somatic symptoms in general practice are more prevalent than anxiety and depressive symptoms (51), the current findings emphasize the fragility of psychosomatic well-being and its dependence on fear of COVID-19 mediated by the destructive power of cyberchondria.

However, these findings contradict the evidence that vaccination against SARS-CoV-2 infection was not predicted by somatic symptoms (52), and persons with high psychosomatic burden are more concerned about their health and visit health care facilities more often (53). Obviously, vaccination against SARS-CoV-2 adherence is more difficult for persons than complying with self-preventive measures. A recent study has shown that vaccine acceptance is positively associated with COVID-19-related anxiety and fears of infection and health-related consequences, whereas social and economic fears are negatively correlated with vaccination willingness (54).

This explains why persons with high socio-economic risks refuse vaccination against SARS-CoV-2. Young persons with high risk for HIV reported wearing facemasks, washing hands, and staying six feet apart, but about one-third of them reported that they would not be vaccinated (55). Refusal or hesitation to vaccinate increased with financial instability (56), working far from the capital (57), lower education background, and uncertainty about the ability of the health care system to treat patients with COVID-19 (58). The non-contributing effect of preventive behavior can be explained by poor knowledge of COVID-19 (59), beliefs in COVID-19 conspiracy theories (60), poor COVID-19 risk perception (61), and skepticism about the virus due to the distrust in politicians and medical scientists (62).

### Limitations and future research

There are several limitations and future perspectives in this study. First, this study was cross-sectional. Previous research suggested that cyberchondria played a moderating role in behavior changes and the growth of concerns regarding the COVID-19 between the first and the second wave of the pandemic (63). To draw conclusions on causal effects, future research must rely on longitudinal data. Second, the findings were obtained in a population sample and cannot be generalized to clinical groups. There is evidence that fear of COVID-19, cyberchondria, and somatization differ between healthy persons and patients with mental and physical diseases (64–66). The patterns found should be examined in clinical groups including patients with somatoform disorders. Third, the relationship between fear of COVID-19 and somatic burden can be mediated by some other characteristics. Future research could explore the relationship between fear of COVID-19, cyberchondria, and psychosomatic burden in a broader biopsychosocial approach. Finally, somatic burden was assessed in this study based on self-report instruments. Although previous research has shown that subjective body complaints can be interpreted as a sign of somatization (67), future studies could examine medical records and health care utilization for a clearer perspective.

## Conclusion

In conclusion, this study contributes to the growing body of knowledge about the relationship between fear of COVID-19 and somatic burden mediated by cyberchondria, but not preventive behavior. It is hoped that changes in the fear of COVID-19 and somatization severity may reduce the burden of cyberchondria, and with low cyberchondria, fear of COVID-19 would not be as destructive to psychosomatic well-being.

## Data availability statement

The original contributions presented in the study are included in the article/supplementary material, further inquiries can be directed to the corresponding author/s.

## Ethics statement

The studies involving human participants were reviewed and approved by Ethical Review Board, HSE University (minutes of the meeting of October 25, 2021). The patients/participants provided their written informed consent to participate in this study.

## Author contributions

The author confirms being the sole contributor of this work and has approved it for publication.

## Conflict of interest

The author declares that the research was conducted in the absence of any commercial or financial relationships that could be construed as a potential conflict of interest.

## Publisher's note

All claims expressed in this article are solely those of the authors and do not necessarily represent those of their affiliated organizations, or those of the publisher, the editors and the reviewers. Any product that may be evaluated in this article, or claim that may be made by its manufacturer, is not guaranteed or endorsed by the publisher.
